# Morphological changes in diabetic kidney are associated with increased *O*-GlcNAcylation of cytoskeletal proteins including α-actinin 4

**DOI:** 10.1186/1559-0275-8-15

**Published:** 2011-09-21

**Authors:** Yoshihiro Akimoto, Yuri Miura, Tosifusa Toda, Margreet A Wolfert, Lance Wells, Geert-Jan Boons, Gerald W Hart, Tamao Endo, Hayato Kawakami

**Affiliations:** 1 Department of Anatomy, Kyorin University School of Medicine, Mitaka, Tokyo 181-8611, Japan; 2 Research Team for Mechanism of Aging, Tokyo Metropolitan Institute of Gerontology, Itabashi, Tokyo 173-0015, Japan; 3 Complex Carbohydrate Research Center, University of Georgia, Athens, GA 30602, USA; 4 Department of Chemistry, University of Georgia, Athens, GA 30602, USA; 5 Department of Biochemistry and Molecular Biology, University of Georgia, Athens, GA 30602, USA; 6 Department of Biological Chemistry, Johns Hopkins University School of Medicine, Baltimore, MD 21205, USA

**Keywords:** *O*-GlcNAc modification, Hexosamine biosynthetic pathway, Kidney, Glomerulus, Cytoskeleton, α-actinin, GK Rat, Mass spectrometry, Proximity Ligation Assay

## Abstract

**Purpose:**

The objective of the present study is to identify proteins that change in the extent of the modification with *O*-linked *N*-acetylglucosamine (*O*-GlcNAcylation) in the kidney from diabetic model Goto-Kakizaki (GK) rats, and to discuss the relation between *O*-GlcNAcylation and the pathological condition in diabetes.

**Methods:**

*O*-GlcNAcylated proteins were identified by two-dimensional gel electrophoresis, immunoblotting and peptide mass fingerprinting. The level of *O*-GlcNAcylation of these proteins was examined by immunoprecipitation, immunoblotting and *in situ *Proximity Ligation Assay (PLA).

**Results:**

*O*-GlcNAcylated proteins that changed significantly in the degree of *O*-GlcNAcylation were identified as cytoskeletal proteins (α-actin, α-tubulin, α-actinin 4, myosin) and mitochondrial proteins (ATP synthase β, pyruvate carboxylase). The extent of *O*-GlcNAcylation of the above proteins increased in the diabetic kidney. Immunofluorescence and *in situ *PLA studies revealed that the levels of *O*-GlcNAcylation of actin, α-actinin 4 and myosin were significantly increased in the glomerulus and the proximal tubule of the diabetic kidney. Immunoelectron microscopy revealed that immunolabeling of α-actinin 4 is disturbed and increased in the foot process of podocytes of glomerulus and in the microvilli of proximal tubules.

**Conclusion:**

These results suggest that changes in the *O*-GlcNAcylation of cytoskeletal proteins are closely associated with the morphological changes in the podocyte foot processes in the glomerulus and in microvilli of proximal tubules in the diabetic kidney. This is the first report to show that α-actinin 4 is *O*-GlcNAcylated. α-Actinin 4 will be a good marker protein to examine the relation between *O*-GlcNAcylation and diabetic nephropathy.

## Introduction

*O*-linked *N*-acetyl-*β*-*D*-glucosamine, termed *O*-GlcNAc, is a post-translational modification involved in modulation of signaling and transcription in response to cellular nutrients or stress by interplay with *O*-phosphorylation [1-3]. *O*-GlcNAc serves as a glucose sensor via the hexosamine biosynthetic pathway. Elevated *O*-GlcNAc modification (*O*-GlcNAcylation) of proteins by increased flux through the hexosamine biosynthetic pathway has been implicated in the development of insulin resistance and diabetic complications and in the up-regulated gene expression of transforming growth factor-beta1, plasminogen activator inhibitor 1, and upstream stimulatory factor proteins in mesangial cells, leading to mesangial matrix expansion and diabetic glomerulosclerosis [2,4-9]. We previously demonstrated increased *O*-GlcNAcylation in the kidney and pancreas of the Goto-Kakizaki (GK) rat, which is an animal model of type 2 diabetes [10,11]. Also, altered *O*-GlcNAcylation and *O*-GlcNAc transferase (OGT) expression were recently reported in the kidney from diabetic patients [12].

In this study we carried out proteomic analysis, especially focused on the proteins with remarkable change of the *O*-GlcNAc level in the kidney from GK rats, and suggested the potential of *O*-GlcNAcylation as a biomarker of diabetic nephropathy. Total kidney proteins from Wistar and GK rats were separated by two-dimensional gel electrophoresis. *O*-GlcNAcylated proteins were detected by immunoblotting using anti-*O*-GlcNAc antibody. Selected proteins that changed markedly in their extent of *O*-GlcNAcylation were identified by Mass Spectrometry (MS) analysis. MS sequencing of tryptic peptides identified some cytoskeletal proteins, including α-tubulin and α-actinin 4. Immunoprecipitation and immunoblot findings demonstrated that *O*-GlcNAcylation of these identified proteins was increased in the diabetic rats. To examine the localization of the identified cytoskeletal proteins, we conducted an immunohistochemical study using confocal scanning microscopy and immuno-electron microscopy. The localization and quantity of these *O*-GlcNAcylated proteins were further examined by performing the *in situ *Proximity Ligation Assay (PLA), which was developed to examine protein-to-protein interaction and post-translational modification of proteins [13,14].

## Methods

### Animals and tissues

Kidney tissues were obtained by dissecting 15-week-old male (n = 3) Wistar rats (as controls) and GK rats, which are a nonobese model of non-insulin-dependent diabetes mellitus and had been developed by the selective breading of glucose-intolerant Wistar rats. Both rats were obtained from CLEA (Tokyo, Japan). All experimental procedures using laboratory animals were approved by the Animal Care and Use Committee of Kyorin University School of Medicine.

### Reagents

Rabbit polyclonal anti-α-actinin 4 antibody was obtained from LifeSpan BioSciences (Seattle, WA). Rabbit polyclonal anti-myosin antibody was obtained from Biomedical Technologies (Stoughton, MA). Rabbit monoclonal anti-actin antibody (clone EP184E) and rabbit monoclonal anti-α-tubulin antibody (clone EP1332Y) were obtained from Epitomics (Burlingame, CA). Mouse monoclonal anti-*O*-GlcNAc antibodies (CTD110.6, 18B10.C7 [3]) were used. The generation of CTD110.6, 18B10.C7(3) was previously described [15,16].

### Two-dimensional gel electrophoresis (2D-PAGE) and immunoblotting

Protein extraction and 2D-PAGE were performed as previously reported [17-19]. Three nondiabetic and 3 diabetic rat kidneys were used simultaneously from protein extraction to gel matching. Five-hundred micrograms of total protein prepared from normal and diabetic kidneys was loaded onto the gel for isoelectric focusing, which was performed by using pre-cast immobilized pH gradient (IPG) strips (18 cm long, pH4-7, GE Healthcare Science). After equilibration in reducing solution and then in alkylating solution, second-dimensional gel electrophoresis was performed by 10% SDS-PAGE. Separated protein spots on polyacrylamide gels were electroblotted onto PVDF membranes. Then total protein spots were stained with BODIPY FL-X (Invitrogen). The membranes were first blocked for 1 h at room temperature with 0.3% BSA in TBS-T and then incubated with mouse monoclonal anti-*O*-GlcNAc antibody (CTD 110.6, Covance) at a dilution of 1:5,000 for 1 hr at room temperature, followed by incubation with biotin-goat anti mouse IgM (Vector) at a dilution of 1:2,000 and then Qdot 625-conjugated streptavidin (Invitrogen) at a dilution of 1:2,000, each for 1 hr at room temperature. The total spots and immunoreactive spots were scanned by using a Molecular Imager FX laser scanning fluorometer (BioRad Laboratories, Hercules, CA). The intensities of spots were analyzed by using PDQuest software ver.8.0 (BioRad Lab). The search for protein spots whose *O*-GlcNAcylation had changed in the GK sample was performed as described previously [17]. The abundance of spots was presented as parts per million of the total spots integrated by using the 'total quantity in analysis set' feature of the PDQuest software. When the abundance of spots on 2D gels of diabetic kidneys was > 2-fold or less than 0.5-fold compared with that for the control nondiabetic kidneys, we regarded the spots as proteins with a changed *O*-GlcNAcylation level.

### In-gel protein digestion and peptide mass fingerprinting

The selected spots were cut from the second dimensional gel stained with SYPRO-Ruby. In-gel protein digestion of selected gel spots was performed according to the protocol described http://www.proteome.jp/2D/2DE_method.html[18]. Peptide-mass fingerprinting data were acquired by using a MALDI-TOF-MS spectrometer (AXIMA-CFR, Shimazu Biotech). Proteins were identified with the Mascot search engine (Matrix Science, London, UK) search algorithms by using the Swiss-Prot protein databases (Version 57.6).

### Immunoprecipitation, immuno-blotting and immunohistochemical study of actin, α-actinin 4, α-tubulin, and myosin

Immunoprecipitation, immuno-blot analysis and immunohistochemical study were carried out as described previously [11].

### In situ PLA analysis

*In situ *PLA analysis was performed according to the manufacturer's instructions with an HRP/NovaRed detection kit from Olink Bioscience (Uppsala, Sweden) [20]. Cryostat sections of kidney were cut and placed onto MAS-coated slides (Matsunami Glass, Tokyo, Japan). The slides were then incubated at 60°C in LAB solution for 5 min. These antigen-retrieved tissues were next washed with PBS, incubated for 15 min in 15 ml/L hydrogen peroxide in PBS, washed, and blocked with 1% BSA-PBS. For the *in situ *ligation assay, we used mouse monoclonal anti-*O*-GlcNAc antibodies (clone: 18B10.C7 [3]). The sections were incubated with this mouse anti-*O*-GlcNAc antibody in combination with rabbit anti-actin, anti-α-actinin 4, anti-α-tubulin or anti-myosin antibody overnight at 4°C. After having been washed with TBS-0.1% Tween20 (TBS-T), the sections were incubated with a mixture of MINUS secondary PLA probe against mouse immunoglobulins and PLUS secondary PLA probe against rabbit immunoglobulins for 1 h at 37°C. Then they were washed with TBS-T, and subsequently incubated in hybridization solution for 15 min at 37°C and washed with TBS-T once for 1 min. The slides were next incubated with the ligation mix for 30 min at 37°C and washed with TBS-T twice for 1 min each time. Then the sections were incubated with the amplification mix for 90 min at 37°C and washed 3 times for 5 min each time with TBS-T. The slides were thereafter incubated with the HRP-labeled hybridization probe for 30 min at room temperature. After 2 washes with TBS for 2 min each time, the slides were incubated with DAB staining mix for 5 min and then washed with water. Nuclei were stained with hematoxyline. The slides were examined with a bright-field microscope equipped with a CCD camera Pro600ES (Pixera, San Jose, CA). In the negative controls in which the primary antibodies had been replaced with normal rabbit IgG and normal mouse IgG or omitted, signals were scarcely observed (data not shown). Signals were quantified with BlobFinderBright software, which is available on BlobFinder Website http://www.cb.uu.se/~amin/BlobFinder/[20].

### Statistics

Data were compiled from 3 independent experiments. Student's t-test was used for statistical analysis, and for all cases P < 0.05 was considered to indicate statistical significance.

## Results

### Identification of O-GlcNAcylated proteins in normal and diabetic kidneys

**Figure 1:**
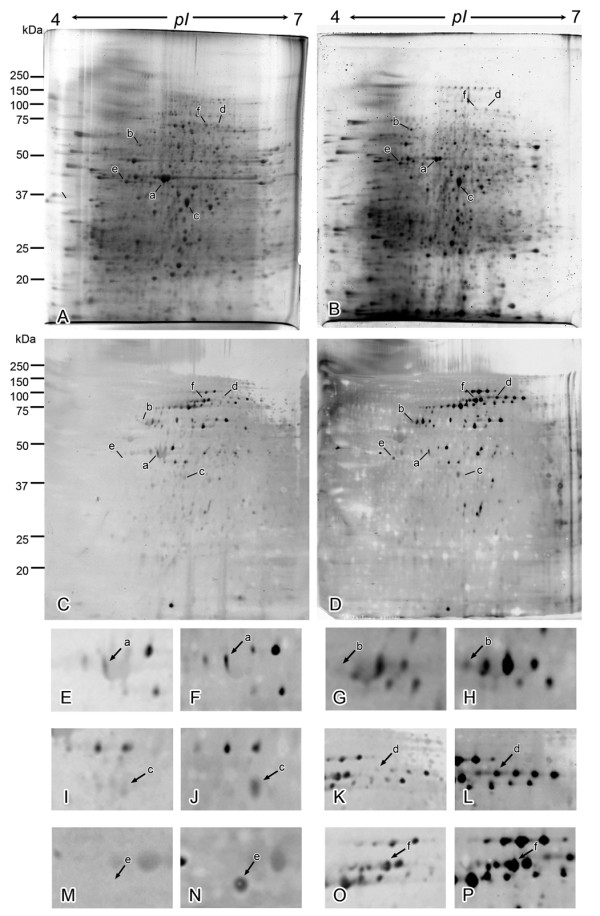
**Comparison of total protein map and *O*-GlcNAcylation map of kidney between nondiabetes and diabetes**. (A, B) Representative total protein map of 2D PAGE for nondiabetic (A) and diabetic (B) rat kidneys detected by SYPRO-Ruby. (C, D) Typical *O*-GlcNAcylated protein map of 2D PAGE for nondiabetic (C) and diabetic (D) rat kidneys detected by *O*-GlcNAc antibody. Spots indicated by arrows represent the identified proteins that had changed in terms of *O*-GlcNAc level. (E-L) Regions around spots "a," "b," "c," "d," "e," and "f" in the maps "C" and "D" are enlarged to facilitate the identification of each spot, indicated by arrows, in the non-diabetic (E, G, I, K, M, O) and diabetic (F, H, J, L, N, P) kidneys.

To identify *O*-GlcNAcylated proteins, we employed two-dimensional electrophoresis and immunoblotting using the CTD110.6 anti-*O*-GlcNAc antibody. Total proteins of diabetic GK rat kidney or nondiabetic Wistar rat kidney were electrophoresed on a two-dimensional gel. Approximately 1,000 protein spots were detected in each SYPRO Ruby-stained gel (Figure 1A, B). Approximately 100 protein spots were detected in each Qdot 625-stained PVDF membrane (Figure 1C, D). The immunofluorescence intensity of each spot was quantified and compared between the nondiabetic kidney and the diabetic one. This comparison revealed enhanced *O*-GlcNAcylation in many proteins from the diabetic kidney (Figure 1C-P). Twenty-seven spots that indicated a significant difference in the relative quantity of *O*-GlcNAcylated protein were applied to MALDI-TOF MS for identification of the proteins. The identified proteins included various cytoskeletal proteins (α-tubulin, α-actinin 4, myosin, actin) and mitochondrial proteins (ATP synthase β pyruvate carboxylase), which were temporarily referred to as spots "a"-"f" (Figure 1, Table 1). The intensity of these spots increased in the diabetic kidneys (Table 1). Almost the same results were obtained among the 3 GK rats as well as the 3 control rats. These proteins except for α-actinin 4 have already been reported to be *O*-GlcNAcylated.

**Table 1:** Proteins showing an increase in the *O*-GlcNAcylation level in the diabetic kidney

Spot	Protein	Theoretical mass (kDa)/pI	Score^(a)^	Peptides	Peptides matched ^(a)^	Fold change^b)^		Sequence
							
				searched		Wistar(control)	GK	Coverage (%)
a	Actin	42.1/5.29	70	20	8	1	2.15	22

b	α-actinin 4	105.0/5.27	118	40	17	1	2.05	21

c	myosin heavy chain	222.5/5.69	54	18	12	1	2.22	9

d	α-tubulin	50.6/4.95	59	51	9	1	2.75	33

e	ATP-synthase β	56.3/5.19	62	27	9	ND^c) ^	↑^d)^	23

f	pyruvate carboxylase	130.4/6.34	73	30	14	1	4.32	16

Spots a-f correspond to those shown in Figure 1.

^a) ^Values of score and peptides matched were determined according to the Mascot search engine on the Swiss-Prot web server (Database: SwissProt 57.6).

^b) ^Fold changes were calculated by using the mean values for the spots of diabetic GK rat kidney relative to those of normal control Wistar rat kidney, which are indicated as "1."

^c) ^ND: not detected.

^d) ^Up-arrow: This *O*-GlcNAcylated spot appeared only in the diabetic kidney.

**Figure 2:**
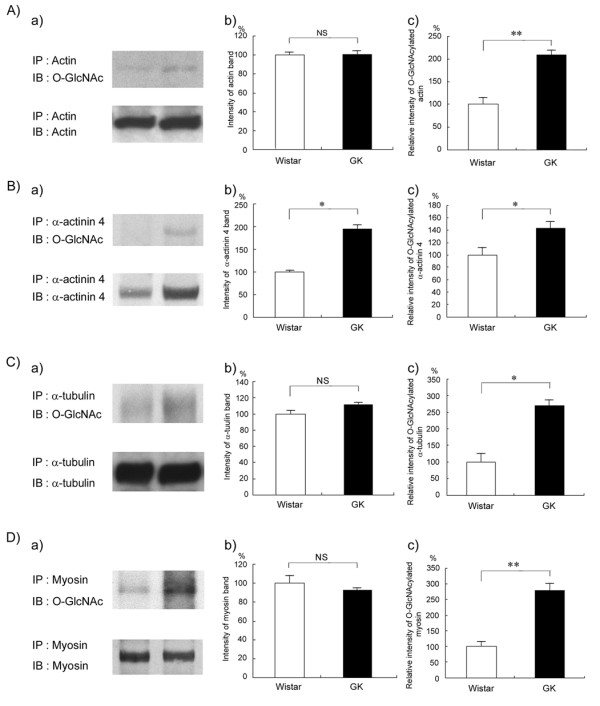
**Analysis of *O*-GlcNAcylation level of cytoskeletal proteins by immunoprecipitation and immunoblotting**. Total lysates of nondiabetic and diabetic kidneys were immunoprecipitated with anti-actin (A), anti-α-actinin 4 (B), anti-α-tubulin antibody (C) or anti-myosin (D). The immunocomplexes were separated by SDS-PAGE, transferred to PVDF membranes, and probed with *O*-GlcNAc antibody (a, upper panel). The membrane was then stripped and reprobed with the antibody used for immunoprecipitation (a, lower panel). Results shown are representative of 3 independent experiments. Intensity of bands recognized by the antibody used for immunoprecipitation was quantified by scanning densitometry (b). Relative intensities of the *O*-GlcNAc reactive bands to bands reactive with each antibody used for immunoprecipitation were then determined (c). Data are the means ± SEM from 3 different rats. (□), Wistar rats; (■), GK rats. *P < 0.05 and **p < 0.01 *vs*. control Wistar rat.

In the next step we focused on the cytoskeletal proteins, as they play important roles in maintaining the morphology of kidney tissue. To further confirm that actin, α-actinin 4, myosin, and α-tubulin were substantially *O*-GlcNAcylated and to examine both the protein level and the level of *O*-GlcNAcylation of these proteins in the diabetic kidney, we performed immunoprecipitation using antibodies against these proteins and compared relative protein expression and *O*-GlcNAcylation by immunoblotting using anti-protein or anti-*O*-GlcNAc, respectively. As shown in Figure 2, the level of these proteins did not change except in the case of α-actinin 4, whose level increased in the diabetic kidney. In contrast, the *O*-GlcNAcylation level of each protein from the diabetic kidney relative to that of each from the nondiabetic one was significantly higher (Figure 2). There might be other proteins in the immunoprecipitants which interacted with or bound to the target proteins. If such proteins were present, it is important to determine the expression level and the *O*-GlcNAcylation level of these proteins between the diabetic and nondiabetic kidney in the future.

### Immunohistochemical study on cytoskeletal proteins

**Figure 3:**
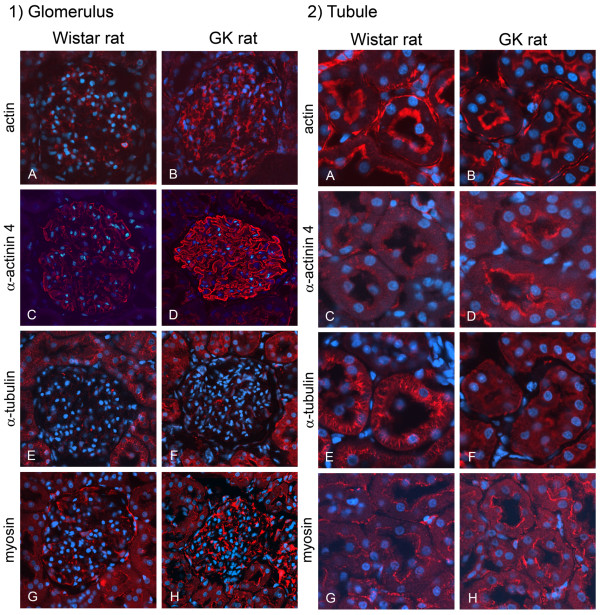
**Immunohistochemical analysis of cytoskeletal proteins by confocal laser scanning microscope**. Localization of actin, α-actinin 4, myosin, and α-tubulin in glomeruli (1) and tubules (2). 1) Although the intensity of immunoreactivity of α-tubulin did not change, that of immunoreactivity of actin, α-actinin 4 and myosin was increased in the diabetic glomerulus. 2) Whereas the intensity of immunoreactivity of actin and myosin did not change, that of immunoreactive α-actinin 4 was increased and that of immunoreactive α-tubulin were decreased in the diabetic tubules. Scale bars, 1)-A, 50 μm, 2)-A, 20 μm.

To examine the localization of the identified cytoskeletal proteins (actin, α-actinin 4, α-tubulin, and myosin), we carried out immunohistochemical analyses of the glomeruli and proximal tubules (Figure 3).

The intensity of immunoreactivity for actin in the glomerulus from the diabetic kidney was increased, especially in its mesangial cells and podocytes (Figure 3B). This result is consistent with an earlier study on the GK rat [21]. The immunoreactivity against actin was also detected in the brush border in the proximal tubules. However, its intensity did not change in the diabetic kidney (Figure 3B)

The immunofluorescence indicating α-actinin 4 was observed as a linear pattern around the lumen of the glomerular capillary, and its intensity in the glomerulus was increased in the diabetic kidney (Figure 3D). α-Actinin 4 was also localized in the brush border area at the luminal side of tubules, and the immunoreactivity was greater in the sections from the diabetic kidney (Figure 3D).

In the glomerulus the immunoreactivity of α-tubulin was weak and did not change in the section from the diabetic kidney (Figure 3F). In the sections showing the proximal tubules of the nondiabetic kidney, a striated immunoreactivity pattern was observed; whereas a diffuse one was noted in the case of the diabetic kidney (Figure 3F).

Whereas weak immunofluorescence was observed in the glomerulus from the normal kidney (Figure 3-1G), intense immunoreactivity was observed in that from the diabetic kidney (Figure 1C-P). Myosin immunoreactivity was also observed in the brush border area of the proximal tubules in sections from both normal and diabetic kidneys, but no difference in intensity was observed between normal and diabetic proximal tubules (Figure 3H).

### Immuno-electron microscopy of α-actinin 4

Because α-actinin 4 has been identified as the causal gene for familial focal segmental glomerulosclerosis and is considered to play an important role in the maintenance of podocyte morphology [22,23], we further examined the precise localization of α-actinin 4 in the glomerulus and tubules by performing immuno-electron microscopy.

**Figure 4:**
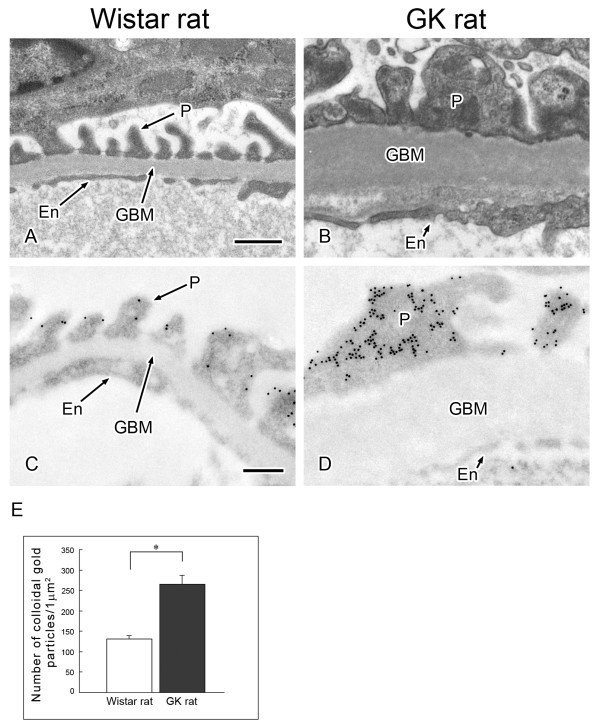
**Morphological change of foot processes of podocytes in glomerulus is correlated with the change of α-actinin 4**. Electron microscopic appearance of glomerular capillary (A, B) and immuno-electron microscopic localization of α-actinin 4 in glomerular capillaries (C, D). (A, B) Comparison of electron micrographs of the capillary wall of the glomerulus from nondiabetic (A) and diabetic (B) kidneys revealed fused foot processes of podocytes and a thickened basement membrane in the sections from the diabetic rat. En, endothelial cell; GBM, glomerular basement membrane; P, foot process of podocyte. Scale bar, 200 nm. (C, D) Sections of nondiabetic and diabetic kidneys were immunolabeled for α-actinin 4 (12-nm gold particles). Whereas the gold particles were localized mainly in the periphery of the foot processes in the nondiabetic kidney, they were found not only in the periphery but also in the inner aspect of foot processes in the diabetic kidney. (E) Density (number/μm^2^) of colloidal gold particles representing α-actinin 4 in the foot processes of podocytes from nondiabetic (open bar) and diabetic (closed bar) kidneys. Data are the means ± SEM from 3 different rats. *P < 0.01 *vs*. control (Wistar rat).

As we had reported previously [10], in the diabetic kidney of the GK rat thickened basement membranes of the capillaries in the glomerulus and fused foot processes of podocytes were observed (Figure 4A, B). Immuno-electron microscopy revealed that in the normal kidney the immunogold particles labeling α-actinin 4 were localized in the cortical area of foot processes of podocytes except beneath the basal plasma membrane (Figure 4C) but that the localization was different in the diabetic kidney; i.e., α-actinin 4 was distributed not only in the cortical area but also in the inner area of foot processes (Figure 4D), and the number of immuno-gold particles was greater in the sections from the diabetic kidney (Figure 4E).

**Figure 5:**
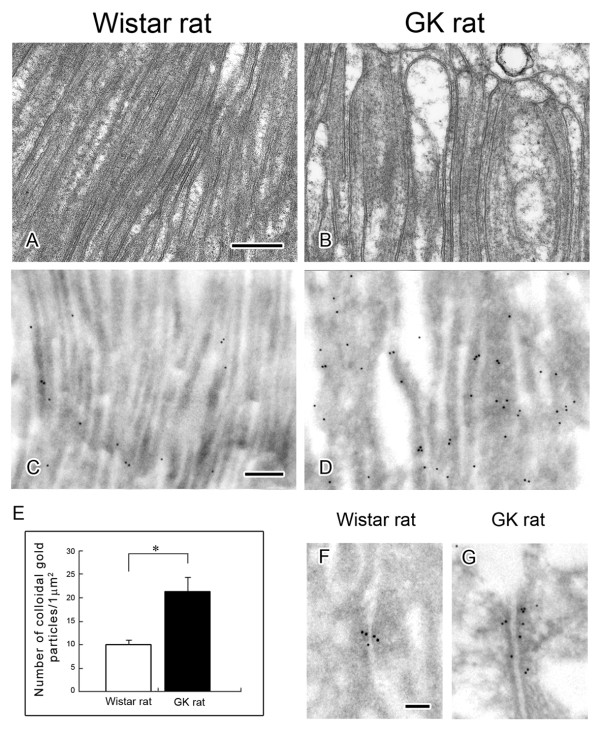
**Morphological change of microvilli in proximal tubule is correlated with the change of α-actinin 4**. Electron microscopic appearance of microvilli of the tubules (A, B) and immuno-electron microscopic localization of α-actinin 4 in microvilli (C, D) and in the adherins junction (F, G) of tubules. (A, B) Comparison of electron micrographs of proximal tubules from nondiabetic rat (A) and diabetic rat (B) revealed that microvilli of the diabetic rat were swollen and had become bulbous, whereas those of the Wistar rat were regularly shaped. (C, D, F, G) Sections of nondiabetic and diabetic kidneys were immunolabeled for α-actinin 4 (12-nm gold particles). Whereas only a few gold particles were localized in the microvilli of proximal tubules in the nondiabetic kidney, many gold particles were detected in the microvilli of proximal tubules in the diabetic kidney (C, D). (E) Density (number/μm^2^) of colloidal gold particles representing α-actinin 4 in the microvilli from nondiabetic (open bar) diabetic kidney (closed bar) kidneys. Data are the means ± SEM from 3 different rats. *P < 0.01 *vs*. control (Wistar rat). (F, G) The colloidal gold labels indicating α-actinin 4 were also observed in the adherins junction of proximal tubules of both nondiabetic (F) and diabetic (G) kidneys. Scale bars: 200 nm (A, C) and 100 nm (F).

Microvilli at the luminal side of proximal tubules from the diabetic GK rats were occasionally swollen and had become bulbous, whereas those from the non-diabetic Wistar rats were regularly shaped and closely packed (Figure 5A, B). Immuno-electron microscopy of sections from the diabetic kidney revealed that colloidal gold particles labeling α-actinin 4 were localized along the full length of the microvilli of proximal tubules of diabetic kidney, whereas in those from the normal kidney the particles tended to be localized near the bottom of the microvilli (Figure 5C, D). The colloidal gold particle density indicating α-actinin 4 in the microvilli was increased significantly in the sections from the diabetic kidney (Figure 5E). The gold label was also observed in the adherence junctions of proximal tubule cells (Figure 5F, G), but no significant difference in the labeling density or localization of α-actinin 4 in these junctions was observed between normal and diabetic kidney sections.

### In situ proximity ligation assay of O-GlcNAcylated cytoskeletal proteins

*O*-GlcNAcylation of proteins is a common type of posttranslational modification. The *in situ *PLA was developed to image protein-to-protein interactions and posttranslational modifications in cells and tissues [13,14]. Using this *in *assay, we examined the localization of *O*-GlcNAcylated cytoskeletal proteins (actin, α-actinin4, α-tubulin, and myosin) and quantified their signals.

**Figure 6:**
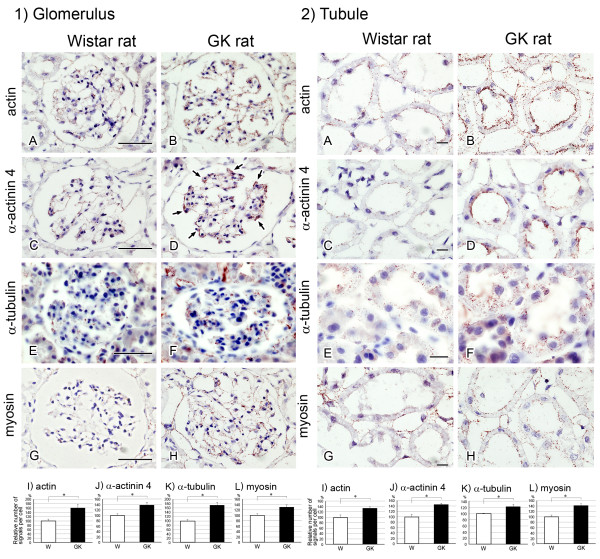
**Analysis of *O*-GlcNAcylated cytoskeletal proteins by using the *in situ *PLA assay**. Localization of *O*-GlcNAcylated actin (A, B), *O*-GlcNAcylated α-actinin 4 (C, D), *O*-GlcNAcylated α-tubulin (E, F) and *O*-GlcNAcylated myosin (G, H) in the glomerulus (1) and tubule (2) of normal (A, C, E, G) and diabetic (B, D, F, H) rats. Arrows in D indicate podocytes. I-L) Relative number of signals per cell. Ten different glomeruli and tubules were obtained from each sample. Signals were analyzed by Blob-Finder software. Values represent means ± SEM from 3 different rats. *P < 0.05 *vs*. control Wistar rat (W).

Signals of *O*-GlcNAcylated-actin, α-actinin 4, α-tubulin, and myosin were observed in the glomerulus (Figure 6-1) and tubules (Figure 6-2) in sections of normal kidney. The number of signals of all these *O*-GlcNAcylated-proteins was significantly increased in both the glomeruli and tubules in sections of the diabetic kidney (Figure,6-1, -2). In the diabetic kidney sections, the signals of *O*-GlcNAcylated α-actinin 4 were increased especially in the podocytes of the glomeruli (Figure 6-1D). The localizations of *O*-GlcNAcylated actin, α-actinin 4, and myosin shown by *in situ *PLA were almost the same as those observed in the conventional immunohistochemical study (Figure 3). However, with respect to the *O*-GlcNAcylated α-tubulin, the striated signal pattern in the normal kidney revealed by the conventional immunohistochemistry (Figure 3-2E) was not observed (Figure 6-2E); rather, the signals were diffusely distributed in the tubules in sections of normal and diabetic kidney (Figure 3F). This observation indicates that polymerized tubulin may not be *O*-GlcNAcylated.

## Discussion

In this study using proteomic analysis, we identified several *O*-GlcNAcylated proteins including cytoskeletal proteins, actin, α-actinin 4, α-tubulin, and myosin and we demonstrated that their extent of *O*-GlcNAcylation was elevated in the diabetic kidney. For the first time, *in situ *PLA studies demonstrated the localization of *O*-GlcNAcylated cytoskeletal proteins and confirmed their increase in *O*-GlcNAcylation.

Actin is an important cytoskeletal protein and is *O*-GlcNAcylated [24]. *O*-GlcNAcylation of actin may modulate the actin-tropomyosin interaction and be involved in the polymerization of myosin heavy chains [24]. High glucose alters protein kinase C-dependent actin assembly in both cultured glomerular mesangial cells and podocytes [25]. Mapping of *O*-GlcNAcylation sites revealed that the Ser^201 ^residue of actin is both *O*-GlcNAcylated and phosphorylated (Table 2) [26,27]. The increased *O*-GlcNAcylation of actin may cause the abnormal assembly of the cytoskeleton, which might have led to the morphological changes in the foot processes and microtubules in the diabetic kidney. It is important to note that if even a small percentage of actin monomers were *O*-GlcNAcylated at a key site regulating filament assembly, this could significantly alter polymerization of the actin network.

**Table 2:** Comparison between phosphorylation sites and *O*-GlcNAcylation sites

Protein	Phosphorylation sites	*O*-GlcNAcylation sites
actin	S35, **S201**^27^	S54, S157, **S201**, S234, S370^26^

α-actinin 4	**Y265**, S423, S621, K625, T667^27, 30^	**S262, S269**, T612, S613, S656*

α-tubulin	Y103, Y224, S232, **Y272**, T334^35, 36^	S6, S158, S178, **T271**, S419**

myosin heavy chain	Y389, Y410, S949, **S1038**, Y1375 Y1415, Y1852, S1956, T1960^39, 40^	S172, S179, S196, S392, S622, S626, S643, S644, S749, S880, **S1038**, S1102, S1148, S1159, S1189, S1200, S1299, S1308, S1336, S1470, S1471, S1596, S1597, S1600, S1606, S1710, S1711, S1777, S1916^26^

Boldface type indicates the site at which *O*-GlcNAcylation and *O*-phosphorylation compete or their respective sites that are close to each other.

* Predicted by YinOYang1.2 prediction server http://www.cbs.dtu.dk/services/YinOYang/

* * Predicted by dbOGAP prediction server https://cbsb.lombardi.georgetown.edu/OGAP.html

The second *O*-GlcNAcylated protein that we identified was α-actinin 4. This is the first report to show that α-actinin 4 is *O*-GlcNAcylated. α-actinin 4 is thought to play an important role in the maintenance of morphology of podocytes by cross-linking with the cortical actin network, which serves as an anchor for a variety of intracellular structures [28,29]. α-actinin 4 is also involved in the bundling of F-actin and vesicular trafficking and has also been identified as the causal gene for familial focal segmental glomerulosclerosis [22]. The present results suggest that abnormal *O*-GlcNAcylation of α-actinin 4 and actin may affect the crosslinking to actin and cause the morphological changes seen in the podocyte foot processes in the glomeruli and in the microvilli of tubules. α-Actinin 4 is reported to be phosphorylated at its Tyr/Ser/Thr residues [27,30], but the *O*-GlcNAcylation sites have not been reported yet. The predicted *O*-GlcNAcylation sites Ser^262 ^and Ser^269 ^are located near the phosphorylation site Tyr^265^, where the phosphorylation at this site regulates the interaction of α-actinin 4 with actin and alters its intracellular location and conformation (Table 2) [31]. Further study remains to be done to clarify the precise *O*-GlcNAcylation sites of α-actinin 4 and their role in the normal and diabetic kidneys.

α-Tubulin is a component of microtubules, which are involved in reabsorption of substances in kidney tubules via the transport of vesicles from the luminal surface to the basal surface of the tubules. It was reported that *O*-GlcNAcylation of miro and milton (OIP106 and GRIF-1) plays a crucial role in the transportation of vesicles and mitochondria via microtubules [32]. The present *in situ *PLA study showed that the *O*-GlcNAcylation of α-tubulin was increased in both the glomerulus and tubule. It has been reported that α-tubulin is *O*-GlcNAcylated [33] and phosphorylated [34-36], but the *O*-GlcNAcylation sites in it have not been determined yet. Thr^271 ^residue, one of the predicted *O*-GlcNAcylation sites, is located next to the phosphorylation sites Tyr^272 ^(Table 2). By inhibiting the phosphorylation of Tyr^272^, *O*-GlcNAcylation of Thr^271 ^may regulate microtubule formation and/or interaction with ligands, such as tau, microtubule-associated proteins 1, 2 (MAPs-1, 2), which are also *O*-GlcNAcylated. Recently it was demonstrated that *O*-GlcNAcylation of tubulin inhibits its polymerization and negatively regulates microtubule formation [37]. The present results suggest that the reabsorption of certain substances in the proximal tubules by transportation via microtubules may be hampered as polymerization of tubulin is inhibited by its abnormal *O*-GlcNAcylation.

Myosin is another important cytoskeletal protein, and it is also *O*-GlcNAcylated [24,38]. *In situ *PLA revealed that the *O*-GlcNAcylation of myosin was increased in both glomeruli and tubules, where myosin plays an important role in the maintenance of the morphology of glomerular cells and microvilli of tubules. Mapping of *O*-GlcNAcylation sites revealed that Ser^1038 ^residue is *O*-GlcNAcylated (Table 2) [26] and this same residue has also been shown to be phosphorylated (Table 2) [39,40]. The role of *O*-GlcNAcylation and phosphorylation of Ser^1038 ^residue remains to be clarified.

In conclusion these results suggest that in the diabetic kidney the morphological changes in the glomerulus and tubules may be ascribed to the abnormal *O*-GlcNAcylation of cytoskeletal proteins including α-actinin 4, which *O*-GlcNAcylation is induced by hyperglycemia-enhanced flux through the hexosamine biosynthetic pathway. α-Actinin 4 will be a good maker to examine the relation between *O*-GlcNAcylation and diabetic nephropathy. *In situ *PLA method could be used for the clinical diagnosis to localize the *O*-GlcNAcylated proteins and quantify them when the antibody against *O*-GlcNAcylated protein is not available. Further studies need to be carried out to clarify the roles of *O*-GlcNAcylation of cytoskeletal proteins in the maintenance of cell morphology and the relationships between *O*-GlcNAcylation of proteins and the etiology of diabetes complications by the glycomic approaches [41].

## List of Abbreviations

*O*-GlcNAc: *O*-linked *N*-acetylglucosamine; *O*-GlcNAcylation: modification of proteins by *O*-GlcNAc; PLA: *in situ *proximity ligation assay; GK rat: Goto-Kakizaki rat; OGT: *O*-GlcNAc transferase; MS: Mass Spectrometry; LAB solution: liberate antibody binding solution; TBS-T: Tris-buffered saline-0.1% Tween 20; MAPs-1, 2: microtubule-associated proteins 1, 2.

## Conflicts of interests

The authors declare that they have no competing interests.

## Authors' contributions

YA performed all experiments, contributed to discussion, and drafted the manuscript. YM, TT and TE performed MS, analyzed data, contributed to discussion and edited the manuscript. HK contributed to discussion and edited the manuscript. MAW, LW, GJB contributed to antibodies and discussion. GWH contributed to discussion, and reviewed and edited the manuscript. All authors read and approved the final manuscript.
